# Ventral hernia repair with a hybrid laparoscopic technique

**DOI:** 10.1111/ans.17508

**Published:** 2022-02-09

**Authors:** Nicholas Bell‐Allen, Kate Swift, Nis‐Julius Sontag, Nicholas O'Rourke

**Affiliations:** ^1^ The Wesley Hospital Brisbane Queensland Australia; ^2^ The Royal Brisbane and Women's Hospital Brisbane Queensland Australia; ^3^ Faculty of Medicine The University of Queensland Brisbane Queensland Australia

**Keywords:** hybrid technique, ventral hernia repair

## Abstract

**Background:**

Ventral hernias are increasingly managed with minimally invasive laparoscopic surgery. Invasive open surgery is typically used for the repair of large‐sized hernias (>10 cm diameter). The two methods are often considered mutually exclusive. We report a hybrid technique for repair of medium to large‐sized hernias.

**Methods:**

Data was collected prospectively from 44 hernias repaired using the hybrid technique from 2012 to 2020. Operative data was examined and follow‐up conducted by both clinical and phone review. As for surgical technique, laparoscopic access was established via a 5 mm optical port and two (or more) 5 mm ports were added under vision. Hernia contents were reduced and extraperitoneal fat excised around the defect. Hernias with diameters ranging from 5 to 10 cm were fixed using the hybrid technique. A small incision was made directly over the hernia and polyester mesh was placed intraabdominally before defect closure with a transfascial suture. Pneumoperitoneum was re‐established and mesh fixation achieved using absorbable tacks and/or fixation sutures.

**Results:**

Of the 44 ventral hernias repaired with the hybrid technique, 43 were secondary hernias from incisional defects. Average hernia diameter was 6.6 cm. 86% of patients were discharged within the first 48 h. Four patients (9%) had recurrences during the study period. Minor complications occurred in 8 patients (18%): 3 (7%) had post‐operative wound infection, 3 patients (7%) developed post‐operative seroma. Two patients (5%) had clinically significant wound haematoma.

**Conclusion:**

Laparoscopic hybrid ventral hernia repair can be safely performed by a combination of laparoscopic and open techniques, offering an alternative method in the management of medium‐sized ventral hernias.

## Graphical abstract

This study describes a hybrid technique combining open and laparoscopic surgery for the repair of medium‐ and large‐sized ventral hernias and demonstrates the results from an 8‐year series of operations performed by one surgeon. We report comparable and occasionally slightly improved outcomes in comparison to the literature. Based on these results, we feel that hybrid ventral hernia repair is safe in comparison to traditional open or laparoscopic techniques for ventral hernia repair.

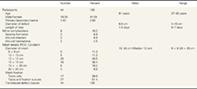

## Introduction

Ventral hernia is a common surgical pathology and incisional ventral hernias complicate 10–20% of laparotomies.^1^ Since its first proposal in 1993, a laparoscopic approach to ventral hernia repair has gradually increased in popularity to replace traditional open repairs. Literature has demonstrated reduced intraoperative bleeding, shorter hospital stay and improved pain scores for the laparoscopic approach.^2^ However, laparoscopy may be complicated in patients with large hernias or adhesions from previous abdominal surgeries. Some evidence suggests that primary fascial closure (PFL) results in reduced seroma formation.^2^, ^3^ To date, there has been no conclusive evidence to suggest hernia recurrence rates are higher with either technique. Nonetheless, both techniques are now widely employed and considered safe.^1^, ^2^, ^3^, ^4^

The choice of operative approach is typically based on defect size, patient factors and surgeon preference. Guidelines published by the International Endohernia Society suggest open repair for primary small defects (<2 cm), and open mesh repair for any large defect (>10 cm) requiring extensive tissue dissection. All other hernias are appropriate for consideration of laparoscopic repair.^3^ Based on these guidelines, open and laparoscopic hernia repair are considered mutually exclusive of one another in most circumstances.

In 2012, we began utilizing a hybrid technique for medium‐ to larger‐sized hernias alongside other centres. The hernia reduction and sac dissection was achieved via a laparoscopic approach, while the defect was then closed via an open incision. We demonstrate that this is a safe and effective procedure for medium‐sized hernias. It benefits from the advantages of laparoscopic surgery and simultaneously limits the peri‐operative complications associated with the larger, more invasive open procedure. This study prospectively analyses a cohort of 44 patients with medium‐sized ventral hernias who underwent elective hybrid repair.

## Methods

Prospective data from surgeries performed by a single surgeon (NO'R) was collected over a period of 8 years (July 2012 to September 2020). Ethical approval was granted by the UnitingCare Health Human Research Ethics Committee. Standard demographic data was recorded together with details about the nature of the hernia (primary versus secondary). Intraoperative details were also collected, including the number of visible hernias, the size of the hernia defect(s), their site on the ventral abdominal wall as well as details on the size of the mesh, number and types of tacks used, and sutures to enhance fixation. The duration of operation, length of hospital stay and immediate peri‐operative complications were documented. All patients were reviewed clinically between 4 and 6 weeks postoperatively. We conducted further follow‐up either by clinical or phone review with a standardized questionnaire.

### Surgical technique

All patients underwent general anaesthetic. Routine use of perioperative antibiotics was employed along with use of a betadine based skin preparation (unless recorded allergy where aqueous chlorhexidine was substituted). Iodine impregnated ‘incise’ drapes were not used. Laparoscopic access was established with the use of a 5 mm Excel Optical trocar (Ethicon, Cincinnati, OH). Optical access was ideally achieved through the upper rectus muscle on the left as tissue planes are more easily defined and the risk of inadvertent visceral injury is reduced. Ultimately, however, access was determined by the site, size and nature of the hernia. Two further 5 mm ports were then inserted under vision on the left anterior axillary line. Sometimes, a 4th or 5th port was required. Laparoscopically, adhesions were divided and hernia contents were reduced. Peritoneum and extraperitoneal fat around the defect was stripped in all directions, ideally with a 3 cm overlap to allow for mesh fixation.

Hernia defects less than 5 cm in diameter were sutured laparoscopically with non‐absorbable barbed suture. Mesh insertion and fixation was achieved via laparoscopic approach, as previously described.^5^ This cohort is excluded from this analysis. Medium sized hernias with defect diameters ranging from 5 to 10 cm were repaired via the hybrid approach. A small incision was made directly over the hernia. The hernial sac was dissected out but there was no lateral subcutaneous dissection of the abdominal fascia, thus reducing the potential subcutaneous space to reduce seroma formation.

Polyester mesh (PCO, Covidien) of appropriate size, aiming for a minimum of 3 cm overlap, was placed into the abdominal cavity via the open defect. The defect was then sutured and closed externally, usually with ‘0’ loop Novofil. Pneumoperitoneum was then re‐established and the previously placed mesh was fixed via a laparoscopic approach to the anterior abdominal wall. Fixation was most often achieved with Absorbatacks (Covidien, Norwalk, CT), or occasionally ProTacks (Covidien, Norwalk, CT). Transfascial fixation sutures were frequently used.

## Results

A total of 44 patients underwent laparoscopically assisted hybrid ventral hernia repair using this method. Twenty‐six patients (59%) were female. Ages ranged from 37 to 82 (mean 61). All but one (98%) of hernias were incisional in nature, with the only primary defect representing a Spigelian hernia.

The mean hernia diameter was 6.6 cm (range 4–10 cm). Fifteen patients (34%) had more than one defect; 14 of which had the largest defect closed using the hybrid technique, with the other smaller adjacent hernias either being incorporated within the single polyester mesh allowing for collective bridging, or requiring a second mesh to attain adequate repair of the satellite hernias. The other patient had two simultaneous hybrid ventral hernia repairs in the same operation. Mesh sizes ranged from 9 × 9 cm circular to rectangular 20 × 25 cm. The mean and median mesh diameters used were 17.4 cm and 12 cm, respectively (Table 1).

**Table 1:** Patient demographics, intraoperative details and post‐operative complications

	Number	Percent	Mean	Range
Participants	44	100		
Age			61 years	37–82 years
Male:Female	18:26	41:59		
Primary:Secondary hernia	1:43	2:98		
Diameter of defect			6.6 cm	4–10 cm
Length of stay			1.5 days	0–7 days
Minor complications	8	18.2		
Seroma formation	3	6.8		
Wound infection	3	6.8		
Wound haematoma	2	4.5		
Mesh details (PCO, Covidien)				
Diameter of mesh			13. 24 cm (Median 12 cm)	9 × 9–20 × 25 cm
9 × 9 cm	5	11.3		
10 × 15 cm	5	11.3		
12 × 12 cm	20	45.5		
15 × 15 cm	16	36.4		
15 × 20 cm	8	18.2		
20 × 25 cm	3	6.8		
Mesh fixation				
Tacks only	17	38.6		
Tacks and fixation sutures	27	61.4		
Transfascial defect closure	44	100		

In 27 patients (71%), the mesh was fixated with both four quadrant fixation sutures as well as absorbable spiral tacks, while the remaining 17 (39%) cases, mesh fixation was achieved with Absorbatacks alone.

There were no documented intraoperative complications, including no mesenteric or enteric injuries during the initial optical access and adhesiolysis. The majority of patients (28 patients, 64%) were discharged within the first 24 h, while only one patient stayed for longer than three nights. The mean length of stay was 36 h (1.5 days).

All patients were reviewed clinically between 4 and 6 weeks postoperatively. Minor complications occurred in eight (18%) patients; three of these were postoperative seromas, none of which had long term clinical implication or morbidity. There were two cases of clinically significant post‐operative wound haematoma, one of which was observed as an outpatient, the other, however, required admission and inpatient management for observation and was covered with IV antibiotics. There were two documented cases of postoperative wound infection. The first of these occurred in a lady on oral immunosuppressants for Rheumatoid arthritis; these medications were temporarily ceased under consultation with her rheumatologist, and the infection was successfully managed as an outpatient on oral antibiotics only. The other was a port site infection for a repair of an intercostal hernia. There are no documented cases of mesh infection, mesh migration or major morbidity in this series.

Follow up ranged from 3 to 79 months (average 27 months). 39 (89%) patients were able to be contacted post their original post‐operative visit in the form of a structured phone interview. Only one of the 44 patients were unhappy with their operation. This was attributed to prolonged (>1 month) post‐operative pain, with no documented hernia on clinical review. When asked, the other 43 (98%) patients said they would return to the operating surgeon should further problems arise.

Assessment for ‘recurrence’ after the original hybrid ventral hernia repair was complex, especially in this series where multiple operations included simultaneous repair of adjacent small to medium sized satellite hernias by mesh coverage only. After ventral hernia repair, patients may develop true recurrence at the edge of the mesh, new hernias distant to the mesh or recurrent mesh lined hernias (a complete bulging or recurrence of the hernia with little residual attachment). For the purpose of this review, all of these ‘categories’ were included as true recurrences. There were four recurrences following hybrid hernia repair in this series, with a documented rate of 9%. 3 (75%) of the recurrences occurred within 12 months of the original hybrid repair, two of which have been subsequently repaired with a laparoscopic approach only, while one required a true open procedure to fix. One is yet to be repaired and is awaiting further follow up. There were no documented port site hernias in this series.

## Discussion

Incisional hernia is a common and potentially morbid complication of abdominal wall surgery. In the literature, recurrence rates are reported as high as 54% when repaired primarily, and 36% when repaired with mesh.^3^, ^4^ The laparoscopic approach to ventral hernia repair has progressively gained popularity as both techniques and equipment evolve. However, laparoscopy may be complicated in patients with large defects or adhesions from previous abdominal procedures.^2^, ^3^, ^4^

There remains no clear conclusion as to whether the laparoscopic approach delivers lower recurrence rates and better outcomes. A meta‐analysis of randomized controlled trials comparing the open versus laparoscopic techniques, published in the International Journal of Surgery in 2015 documented no statistically significant difference in hernia recurrence rates. Nonetheless, there was a significantly reduced rate of wound infection in the laparoscopic group.^4^ Furthermore, Sajid *et al*. documented better short‐term outcomes for the laparoscopic group, including shorter hospital stay and better pain tolerability.^6^

A hybrid approach to ventral hernia repair has recently been introduced, with promising outcomes. Recent publications are now concluding that combining PFL with mesh fixation reduces both seroma rate and recurrence.^7^, ^8^ Additional putative benefits of this technique and its more robust means of mesh fixation, include the reduction in the chance of mesh migration and ‘ballooning’, both of which predispose patients to actual and/or presumed recurrence.^5^, ^9^ Moreover, Bernardi *et al*. have recently published in the Annals of Surgery, concluding that defect closure during laparoscopic ventral hernia repair results in statistically significant improvement in patient quality of life (QoL) outcomes; including pain, cosmesis, function and overall satisfaction.^10^ The ability to excise old scars is an extra benefit; especially in patients where unappealing deformities have resulted, particularly in the context of previous wound infection. We feel, for selected patients, combining both techniques allow for the aforementioned benefits of laparoscopic surgery, whilst reducing the risk of seromas and infection that may follow extensive soft tissue dissection and its sequelae required from a purely open technique. Moreover, the initial laparoscopy, as in our approach, allows the surgeon to identify additional occult or sub‐clinical hernias that may have otherwise been missed but can thereafter be repaired, leading to a reduction in not only recurrence, but also new hernia development.^11^ Ji *et al*. a Chinese group, were one of the first to publish a proposed hybrid repair in 2012, which would combine the benefits of minimally invasive surgery while allowing open access and preventing prolonged complex laparoscopic repair.^12^ They compared patients requiring early versus late conversion from a laparoscopic to open repair due to technical difficulty, revealing that early conversion resulted in decreased operative time, reduced hospital length of stay, and less iatrogenic enterotomy. This suggests that in complex repairs, an early or planned combined approach may have better outcomes than a complex laparoscopic‐assisted repair requiring open conversion. Their most common indications for conversion were difficult adhesiolysis, suspected bowel injury, or difficult hernia reduction.^12^

More recently, in 2019, a prospective randomized controlled trial from Ahonen‐Siirtola *et al*., a Finnish group, was published in *Surgical Endoscopy* comparing the traditional laparoscopic ventral hernia repair versus the hybrid approach at the year 1 mark.^13^ This included 82 patients in the hybrid group with a mean hernia diameter of 10.5 cm. The recurrence rate at follow up in both the laparoscopic and hybrid group was 7% and 6%, respectively, a more realistic comparison to our documented recurrence rate. In our series, recurrence was assessed by a combination of clinical and phone review. Admittedly, some recurrences may have been missed as 11% of patients were uncontactable beyond 12 months. Of those who received follow up (mean 27 months), however, it is unlikely that many patients would experience late recurrence as the literature indicates that recurrences often occur during the first 2 years after repair, as is consistent with our findings.^8^, ^14^ We were, however, very liberal in assessing and including ‘recurrences’ in our series to ensure transparency in our data. Certainly, at least 2 of our documented recurrences could be considered new hernias, distant to the mesh placed at the original hybrid repair (Table 2). This would significantly lower our recurrence rate to under 5%. To reduce recurrence, our series aimed for mesh overlap of at least 3 cm. As this was achieved laparoscopically with tacks, there did exist a tendency for the mesh to be pushed away from the tacker potentially leaving asymmetrical coverage and a heightened risk for recurrence at the edge of the mesh. Only one of the four recurrences was documented when fixation sutures were used with Absorbatacks.

**Table 2:** Details of the hernia recurrences in 4 of 44 patients (9%)

Patient age and sex	65 year‐old F	62 year‐old M	67 year‐old M	48 year‐old M
Original defect diameter	8 cm	3 defects, largest 5 cm	6 cm	3 defects, 4 cm each
Hernia type	Incisional ventral	Incisional epigastric	Supraumbilical	Recurrent periumbilical
Mesh fixation technique	Tacks only	Tacks only	Tacks only	Tacks and fixation suture
Mesh size	9 × 9 cm	10 × 15 cm	12 × 12 cm	10 × 15 cm
Type of recurrence	Site of mesh	Edge/new distant hernia	Edge/new distant hernia	Edge/new distant hernia
Time to recurrence	<12 months	6 months	24 months	8 months
Recurrent hernia repair	Open repair with on‐lay mesh	Laparoscopic repair	Laparoscopic repair	Not yet repaired

Post‐operative pain was not an issue for the large majority of our patients. Pain scores were not formally recorded, however, 15 out of 32 patients (47%) had no narcotic analgesia requirement at time of hospital discharge. Pain reducing methods in our series included peritoneal stripping laparoscopically, limited open soft tissue dissection, as well as liberal use of non‐steroidal suppositories. Research is lacking in post‐operative pain outcomes for patients undergoing laparoscopic or open hernia repairs, and this could be improved with numeric pain rating scale or visual analogue scale at intervals post‐op, or a QoL questionnaire. There is a paucity of evidence to suggest that laparoscopic repair causes less pain than open repair, or that 10 mm ports cause more pain than 5 mm ports.^15^, ^16^

Recent studies have demonstrated that closing the hernia defect reduces the incidence of seroma.^3^, ^17^ Our seroma rate was 6.8% (3 out of 44 patients) with no associated long‐term morbidity, lower than the 16% seroma rate in the literature.^13^ We feel that our seroma rate was low because the hybrid approach allows minimal dissection of the tissues around the hernial sac. Seroma is not uncommon with on‐lay meshes and although it usually resolves spontaneously, it can be annoying for patients and occasionally requires re‐intervention.

Our infection rate was 6.8% (3 out of 44 patients), all of which were superficial with no mesh involvement. This is comparable to the reported infection rate for laparoscopic repair in the literature, which ranges from 1.8% to 12%.^1^ Open repairs, however, with the inherent necessity for more extensive tissue dissection and manipulation, generate higher wound infection rates.^4^ By using this hybrid technique, we attempt to reduce this by limiting open soft tissue dissection. This is further supported by the use of smaller 5 mm ports, which are known to have much smaller rates of port site hernia recurrence or infection when compared to larger ports.^18^

Given that our series demonstrates similar low rates of complications and recurrence to other recent reports, we propose that the hybrid approach to ventral hernia repair is a safe and reasonable approach for medium‐sized hernias.

## Conflict of interest

None declared.

## Author contributions

**Nicholas Bell‐Allen:** Data curation; investigation; project administration; visualization; writing – original draft; writing – review and editing. **Kate Swift:** Investigation; visualization; writing – original draft. **Nis‐Julius Sontag:** Visualization; writing – original draft. **Nicholas O'Rourke:** Conceptualization; methodology; project administration; supervision.
